# Hypersplenism in liver disease and SLE revisited: current evidence supports an active rather than passive process

**DOI:** 10.1186/s12878-016-0042-z

**Published:** 2016-02-09

**Authors:** John M. Gemery, Andrew R. Forauer, Anne M. Silas, Eric K. Hoffer

**Affiliations:** Division of Interventional Radiology, Department of Radiology, Dartmouth-Hitchcock Medical Center, One Medical Center Drive, Lebanon, NH 03766 USA; Geisel School of Medicine at Dartmouth, One Rope Ferry Road, Hanover, NH 03755 USA

**Keywords:** Hypersplenism, Liver disease, Systemic lupus erythematosus, SLE, Hematopoiesis, Cytopenias, Splenomegaly, Stem cells, Doan, Dameshek

## Abstract

**Background:**

Active and passive theories have been advanced to explain splenomegaly and cytopenias in liver disease. Dameshek proposed active downregulation of hematopoiesis. Doan proposed passive trapping of blood components in a spleen enlarged by portal hypertension. Recent findings do not support a passive process.

**Discussion:**

Cytopenias and splenomegaly in both liver disease and systemic lupus erythematosus (SLE) poorly correlate with portal hypertension, and likely reflect an active process allocating stem cell resources in response to injury. Organ injury is repaired partly by bone-marrow-derived stem cells. Signaling would thus be needed to allocate resources between repair and routine marrow activities, hematologic and bone production. Granulocyte-colony stimulating factor (G-CSF) may play a central role: mobilizing stem cells, increasing spleen size and downregulating bone production. Serum G-CSF rises with liver injury, and is elevated in chronic liver disease and SLE. Signaling, not sequestration, likely accounts for splenomegaly and osteopenia in liver disease and SLE. The downregulation of a non-repair use of stem cells, bone production, suggests that repair efforts are prioritized. Other non-repair uses might be downregulated, namely hematologic production, as Dameshek proposed.

**Summary:**

Recognition that an active process may exist to allocate stem-cell resources would provide new approaches to diagnosis and treatment of cytopenias in liver disease, SLE and potentially other illnesses.

## Background

In the 1940s and 1950s there were two competing theories to account for the coexistence of cytopenias and splenomegaly. Charles Doan proposed a passive process, splenic sequestration, whereby increased portal pressure causes an enlarged spleen that traps blood components, creating cytopenias. William Dameshek postulated an active process, in which a signal, possibly produced by the spleen, downregulated hematopoiesis [1, 2]. Doan’s theory became dominant as early as the 1960s, and even Dameshek is alleged to have said, “Well, it looks as though Charley Doan is right” [3].

In light of current knowledge, Dameshek may have conceded too soon. At least in liver disease and systemic lupus erythematosus (SLE), splenomegaly and cytopenias are poorly correlated with portal hypertension, and there is evidence for downregulation of hematopoiesis, as Dameshek suggested. Splenomegaly and cytopenias, at least to some degree, likely reflect an active process that mobilizes bone marrow stem cells and allocates their distribution among competing demands in response to injury.

## Discussion

### Active and passive theories: signaling or sequestration?

The theory of hypersplenism is based on the idea of splenic sequestration. Liver damage impedes portal venous inflow, causing elevated portal venous pressure and, in theory, an enlarged spleen. The enlarged spleen is alleged to sequester blood components, causing cytopenias. The theory thus accounts for both the splenomegaly and cytopenias found in association with liver disease [4].

Portal hypertension leading to splenic enlargement and sequestration is not, however, a clean explanation of the cytopenias associated with liver disease. Despite the suggested causal relation, spleen size does not correlate with portal pressure [5]. Furthermore, enlargement of the spleen alone may not be sufficient to create cytopenias. For example, pharmacologically increasing the size of the spleen does not result in a corresponding decline in platelet count [6, 7].

A patent surgical splenorenal shunt or a transjugular intrahepatic portosystemic shunt (TIPS) will reduce portal venous pressure, yet will neither resolve pre-existing cytopenias nor prevent the development of new cytopenias [8, 9]. This lack of response could still be explained by hypersplenism if spleen size were to become fixed once enlarged, but this does not appear to happen. Both spleen size and thrombocytopenia correct after liver transplantation, but thrombocytopenia may resolve months prior to significant change in spleen size [10]. Hypersplenism has also been advanced to account for splenomegaly and cytopenias in (SLE), yet liver disease and thus portal hypertension are uncommon features of SLE [11, 12]. In all, there is poor correlation between spleen size, portal hypertension and cytopenias in both liver disease and SLE.

Other factors that potentially contribute to cytopenias in liver disease have been proposed, including insufficient thrombopoietin (TPO) production by the liver, and marrow injury due to hepatitis viruses, alcohol abuse and poor diet [4]. There are, however, confounding observations which cast doubt on these possibilities.

TPO levels in liver disease appear to be variable, and lack of TPO does not suggest a reason for splenomegaly nor for cytopenias other than thrombocytopenia [13–16]. Nutritional optimization does not prevent the development of cytopenias [17]. Viral or alcohol marrow injury does not explain the cytopenias of patients with liver disease due to autoimmune hepatitis or cystic fibrosis [18–21]. Additionally, patients who have cytopenias before liver transplant are usually able to correct those cytopenias after transplant using the same bone marrow [22–24].

### G-CSF: an alternative explanation

An alternative explanation is that splenomegaly in liver disease and SLE is part of a response to increased demand for stem cells needed for organ repair and triggered by a rise in serum G-CSF. G-CSF administration mobilizes stem cells from marrow with a side effect being enlargement of the spleen [6]. Serum G-CSF has been found to rise with liver injury and to be elevated in both liver disease and SLE [25–28].

In addition to splenomegaly and cytopenias, liver disease and SLE are also characterized by the development of osteopenia that is not explained by hypersplenism [29, 30]. G-CSF, in addition to its effect on stem cell mobilization, also downregulates bone production—and thus potentially provides a unified explanation for splenomegaly and osteopenia [31]. Since increasing spleen size alone does not result at least in thrombocytopenia [6], some additional effect(s) must occur to account for cytopenias.

Downregulation of bone production by the G-CSF mediated process of stem cell mobilization suggests that repair efforts take precedence over the routine marrow activity of bone production. If so, then downregulating hematopoiesis along with bone production would be logical and exactly what Dameshek proposed well before the discovery that bone-marrow-derived stem cells repair damaged organs. Clinical observations and experimental work indicate that bone marrow stem cell resources are finite; thus, allocation between competing uses would be needed.

In the years since Doan’s theory superseded Dameshek’s, there have been reports of inhibition of bone marrow culture by sera from cytopenic SLE patients and from cirrhotics, as Dameshek had postulated [32, 33]. At the time of their publication, these reports did not, however, lead to a re-evaluation of Dameshek’s theory. The finding that offers a logical explanation for hematopoietic downregulation had not yet been made; namely, that bone marrow stem cells contribute to the repair of the liver and other organs.

### Repair by stem cells

The discovery that liver injury is repaired in part by bone-marrow-derived stem cells has shown that in liver injury there is a demand for stem cell resources [34]. Eckersley-Maslin et al., reviewing human and animal studies, noted that both the duration and severity of liver injury were positively correlated with the degree of incorporation of bone-marrow-derived cells into the liver [35]. More severe and chronic liver injury will place more demands on bone marrow stem cell resources.

The mobilization of stem cells from bone marrow involves cytokine signaling including G-CSF. Clinically, G-CSF is used to mobilize stem cells to allow collection, via apheresis, of sufficient numbers for bone marrow stem cell transplantation. One of the side effects of G-CSF administration is enlargement of the spleen. The G-CSF dose is limited to minimize the risk of over-enlargement of the spleen and splenic rupture, which is a reported complication [6].

As administered G-CSF communicates demand for stem cells in bone marrow transplant donors, so may endogenous G-CSF in liver injury. Lemoli et al. noted a significant increase in serum G-CSF following liver injury (i.e., both liver resection and hepatic transplantation), with a corresponding increase in the number of stem cells in circulation. Non-hepatic abdominal surgery did not show a similar increase in serum G-CSF [25].

The rise in serum G-CSF does not appear to be restricted to hepatic injury in the form of surgery. Stoiser et al. found significantly increased serum G-CSF in patients with acute malaria who had evidence of concurrent liver injury with elevated serum bilirubin and alanine transaminase (ALT). The effect also does not appear to be limited to acute liver injury [36]. Kaya et al. reported serum G-CSF to be significantly higher in patients with cirrhosis than in normal control subjects, as well as reporting a trend toward higher serum G-CSF levels with increasing Child-Pugh classification [27]. A potentially confounding variable is that all patients studied by Kaya et al. [27] had hepatocellular carcinoma (HCC), which has been reported to produce G-CSF in some instances [37]. Bazarniy et al., however, have also reported increasing serum G-CSF corresponding to worsening Child-Pugh scores in patients with cirrhosis, who were not known to have HCC [28].

Splenomegaly is thus more consistently associated with increased serum G-CSF than with elevated portal venous pressure. G-CSF both mobilizes stem cells and downregulates bone production. Downregulation of bone production, and potentially hematopoiesis, in conjunction with stem cell mobilization would not seem logical unless bone marrow stem cell resources were insufficient to meet all demands simultaneously.

### Finite stem cell resources?

Animal experiments and clinical studies have raised the possibility that bone marrow stem cell resources are finite and that insufficient stem cell resources may manifest as cytopenias. The concept of finite stem cell resources has been incorporated into clinical practice in the timing of chemotherapy and bone marrow stem cell collection for autologous stem cell transplantation. Stem cell mobilization is intended to allow collection of sufficient numbers of stem cells for successful transplantation, but this does not work in all cases. Prior chemotherapy is associated with insufficient stem cell collection, which researchers believe is due to the toxicity of chemotherapy to stem cells [38, 39]. To minimize the risk of insufficient collection, stem cell mobilization and collection are performed earlier, rather than later, in a course of chemotherapy [38].

In addition to prior chemotherapy, platelet count at the time of mobilization is a significant predictor of stem cell yield, with lower platelet counts associated with insufficient collection [38]. A history of prior chemotherapy and a lower platelet count would appear to convey the same information, namely that there are fewer stem cells available. The cytopenias of liver disease may indicate the same deficiency.

Animal experiments are also consistent with reduced stem cell resources manifesting as cytopenias. Seed et al. studied beagles exposed to gamma radiation for 22 hours per day for life with different groups receiving different exposure rates. Platelet and leucocyte counts declined with initial irradiation, but then plateaued. The higher the daily radiation exposure, the lower the plateaus. Despite the ongoing irradiation, the plateaus were generally maintained over an initial 1,000-day observation period. Erythrocyte counts were more resistant to radiation dose and did not decline in the lower exposure groups but did at higher levels [40].

The pattern suggests a process whereby production of erythrocytes is maintained, in preference to platelets and leucocytes, as marrow resources are diminished. The appearance of cytopenias in liver disease also appears to conserve erythrocyte production in preference to platelets and leucocytes. Qamar et al. found that during follow-up (median 54.9 months), patients with compensated cirrhosis developed thrombocytopenia at a median of 28 months, leucopenia at a median of 30 months and anemia at a median of 39.6 months [41]. The similarity in development of cytopenias between irradiated dogs and cirrhotic humans may reflect the same underlying state, namely insufficient marrow stem cell resources. Whether diminished by radiation or diverted to repair, there appear to be insufficient marrow stem cells to maintain normal hematologic production.

### Cytopenias and risk of death

A similar pattern of mortality in irradiated dogs and cirrhotics is also consistent with diminished stem cell resources in liver disease. In irradiated dogs, the risk of death increased with the degree of cytopenias [40]. In cirrhotic patients, the risk of death also corresponded with the severity of cytopenias. Qamar et al. noted that patients without hematologic abnormalities had a 6 % mortality rate during their study vs. 18 % for those with thrombocytopenia and 28 % for those with both thrombocytopenia and leucopenia [41].

If insufficient stem cell resources result in cytopenias, then we might expect a reduced demand for stem cell resources to allow resumption of normal hematologic production and correction of cytopenias. For example, if freed from the need to support an injured liver, stem cell resources might resume hematologic production. Liver transplantation does reverse pre-transplant cytopenias in most instances, and can do so months prior to any significant decrease in spleen size [10, 22]. Of note, cases where there is delay in return to normal hematologic parameters after transplant may occur in patients who had poor stem cell resources prior to transplant, and possibly a higher demand for stem cell resources after transplant.

Stanca et al. investigated the persistence of thrombocytopenia after liver transplantation and found that patients with persistent thrombocytopenia had significantly lower platelet counts (*P* < .001) prior to transplant, and had received livers from older donors (*P* < .04). No significant differences were noted in recipient age, etiology of liver disease, bilirubin level, international normalized ratio (INR), Mayo end-stage liver disease (MELD) score, United Network for Organ Sharing (UNOS) status, or other donor variables. The lower pre-transplant platelet count suggests, as in autologous stem cell transplant patients, reduced stem cell resources. The older donor livers may require more stem cell support from recipients who are already “stem cell poor” [42].

Observations in patients with liver disease do match those of patients and experimental animals with reduced stem cell resources. If stem cell resources were, in fact, finite, then managing allocation between completing uses would require signaling for allocation between competing demands. Signaling, such as Dameshek proposed, would not be needed in splenic sequestration, as that process would be passive.

### Signaling?

There is direct evidence of in vivo downregulation of a non-repair demand for stem cell resources, namely bone production. Bone abnormalities in patients with liver diseases, termed hepatic osteodystrophy, are well described. In a review article, Luxon noted that osteoporosis is common in patients with disparate liver diseases, that the etiology is poorly understood, and that the prime cause appears to be decreased bone formation rather than increased resorption [29].

Elevated serum G-CSF may provide the explanation, downregulating bone production while mobilizing stem cells for repair efforts. Long-term administration of G-CSF is associated with the development of osteopenia in humans, and causes bone loss in mice [31, 43].

If bone production is downregulated, then other non-repair uses for stem cells might be as well, namely hematologic production, as Dameshek suggested. Ohki et al. reported that sera from patients with cirrhosis and anemia, when added to cultures of normal marrow, suppressed colony formation of hematopoietic progenitor cells. The degree of suppression correlated with the severity of patient cytopenia. Sera from patients who were cirrhotic but not anemic did not cause suppression [32]. These results were published in 1988, well after Doan’s theory superseded Dameshek’s, and well before the discovery that bone-marrow-derived stem cells incorporate into the liver. (Note: Ohki’s study employed the marrow culture techniques available at the time—using agar as a culture medium—and to date has not been repeated using current bone marrow culture techniques that employ methylcellulose as a culture medium.)

In addition to granulocyte-colony stimulating factor (G-CSF), Interleukin 17 (IL-17) and Interleukin 23 (IL-23) are critical components of stem cell mobilization, at least in mice. Mice with a genetic defect resulting in chronically elevated serum G-CSF display higher than normal numbers of circulating stem cells. Additionally, these mice have high serum levels of IL-17 and IL-23. Antibody blocking of any one of the three cytokines reduces stem cell mobilization [44]. G-CSF, IL-23, and IL-17 have all been found to be elevated in diverse liver injuries, consistent with activation of the stem cell mobilization process [27, 28, 45–49]. Blocking or reducing any of the three cytokines may have the same effect in humans as it does in mice, reducing stem cell mobilization.

Splenic embolization has been employed to improve cytopenias in patients with liver disease [50, 51]. The at least partial success of this approach might appear to support the theory of splenic sequestration. The effect may instead be due to altered signaling and provide further support for Dameshek’s theory.

In mice, IL-23 is produced in part in the spleen [44]. Splenic embolization in patients with liver disease may in fact be reducing IL-23 production, and thus the mobilization of marrow resources for repair, leaving marrow stem cell resources in place for hematologic production.

The above in combination suggest not the passive process of splenic sequestration promoted by Doan, but rather, the active management of stem cell resources, employing signaling, including the downregulation of hematopoiesis suggested by Dameshek. A management process diverting bone marrow stem cell resources away from hematologic and bone production, and toward hepatic repair, could thus account for splenomegaly, cytopenias and loss of bone mass observed in patients with liver disease.

### Systemic Lupus Erythematosus (SLE)

If there were a mechanism that managed stem cell resources, that mechanism ought to be active not solely in liver disease, but also in other situations where there are competing demands for stem cell resources. Bone-marrow-derived cells can, in fact, incorporate into tissues other than the liver [52], raising the possibility that repair efforts in other disease states could trigger the active allocation of stem cell resources.

With activation of a stem cell allocation mechanism, one might anticipate findings that are due to allocation rather than to the injury that is triggering the allocation process. If the allocation process is active in liver disease, then other disease states that activate the same allocation mechanism might share similar findings: namely, splenomegaly; progressive hematologic abnormalities with associated mortality; osteopenia; evidence of stem cell mobilization signaling, such as elevation of serum G-CSF; and possibly suppression of hematopoiesis. We looked for other disease states that have chronic injury and thus might have activation of a stem cell allocation mechanism. Patients with (SLE) suffer chronic multi-organ injury and have findings matching the above [11, 26, 30, 33, 53–55].

Hematologic abnormalities are common in patients with SLE, and include anemia, leucopenia and thrombocytopenia [11]. Cytopenias in SLE are associated with greater disease activity and greater mortality, paralleling the increase in mortality with worsening cytopenias reported in patients with liver disease [41, 53, 56]. There are multiple proposed etiologies for the cytopenias, including immune and non-immune processes and, as in patients with chronic liver disease, splenic sequestration [11]. Liver disease, and thus portal hypertension, is, however, infrequent in SLE [12].

Splenomegaly does occur in SLE, and may be due to a response to demand for stem cell resources rather than portal hypertension. Hellmich et al. found that patients with SLE and neutropenia had a mean serum G-CSF level more than double that of SLE patients without neutropenia (*p* = 0.007) [26]. Elevation of serum G-CSF suggests, as in liver disease, both a demand for, and mobilization of, stem cells, with splenomegaly a consequence.

More extensive injury could be expected to generate greater demand for stem cell resources for repair. Splenomegaly in SLE patients does correlate with SLE disease activity, in that patients with more severe disease were found to have larger splenic volumes [55].

Again paralleling liver disease, patients with SLE may develop osteoporosis. The elevated serum G-CSF noted by Hellmich might contribute, at least in patients with sufficient stem cell demand to have chronic elevation of serum G-CSF [26]. In fact, Pineau et al. found that SLE patients with osteoporosis had significantly longer disease duration and greater SLE-related organ damage than those with normal bone density [30]. The lower bone density did not correlate with steroid use. It is thus possible that chronically elevated G-CSF in these individuals may result in loss of bone mass.

There may be downregulation of hematologic production in SLE as in liver disease. Pyrovolaki et al. have reported upregulation of apoptosis of hematopoietic progenitor cells in SLE patients [57]. Dainiak et al. noted suppression of hematopoietic precursors in bone marrow culture by SLE sera [33], paralleling the suppression of hematopoietic precursors in marrow culture by cirrhotic (and anemic) sera reported by Ohki [32]. The effect appears to be reversible; Dainiak reported a patient whose sera obtained during an SLE flare suppressed marrow culture, while sera obtained during a remission did not [33]. SLE can be treated with bone marrow transplantation where reduced disease activity (less auto injury) might result in reduced demand for stem cell resources for repair. Wang D. et al. reported significant increases in hemoglobin and platelet count in SLE patients following allogeneic mesenchymal stem cell transplantation [58].

Irradiated dogs and patients treated with chemotherapy have cytopenias attributable to reduced bone marrow stem cell resources. We have suggested that the cytopenias in liver disease may also reflect limited stem cell resources, due not to destruction of stem cells, but rather to diversion of stem cells to repair efforts. Cytopenias in patients with SLE may also reflect reduced stem cell resources due to repair efforts. Statkute et al. reported that SLE patients had significantly lower stem cell mobilization than patients with multiple sclerosis treated with the same mobilization regimen [59].

## Summary/Conclusions

The contribution of bone marrow stem cells to the repair of organs was unknown at the time when Doan and Dameshek developed their theories to account for the association between liver disease, splenomegaly and cytopenias. The discovery that bone marrow contributes to organ repair has two relevant consequences. One, that there is a normal sequence of events in a response to a demand for stem cells; and two, that there may be competition for stem cell resources in the event of an injury.

The normal response to stem cell demand appears to include splenic enlargement as demonstrated in bone marrow stem cell donors, where G-CSF administration both mobilizes stem cells and causes splenic enlargement. That enlargement is not accompanied by a drop in platelet count, and in fact white blood cell counts increase, suggesting that cytopenias are not due to spleen size alone nor to G-CSF directly [6, 7]. The disconnect between spleen size and cytopenias is supported by the observation that splenomegaly persists after correction of cytopenias following liver transplant [10]. Additionally, spleen size does not correlate with portal venous pressure nor does decompression of the portal venous system either correct or prevent cytopenias [5, 8, 9]. These observations raise doubts as to sequestration being a full explanation of cytopenias in liver disease.

If spleen size is not altered by portal pressure in liver disease, then by what is it altered? The elevated serum G-CSF reported in two studies of patients with liver disease suggests an alternative, that splenic enlargement in liver disease is, as in bone marrow donors, part of the normal response to demand for stem cells [27, 28]. Concurrent splenomegaly and liver injury thus more likely reflect response to injury, rather than Doan’s theory that increased portal venous pressure causes splenic enlargement and sequestration. Organ injury, splenomegaly and elevated G-CSF are also present in SLE, typically without liver disease [12, 26, 55]. This is further evidence in favor of splenic enlargement being a component of a response to injury, possibly mediated by G-CSF, rather than an effect of portal pressure. If increased spleen size is a part of a normal response to demand for stem cells, and is not accompanied by cytopenias in normal individuals (bone marrow donors), then why do cytopenias occur in patients with liver disease or SLE?

Injury that triggers stem cell demand for organ repair will increase the total demand for bone marrow stem cells. Unless stem cell resources are infinite, this added demand might result in a need for prioritization. Experience with cancer patients and irradiated dogs indicates that stem cell resources are not infinite, and that low stem cell resources may be evidenced as cytopenias [38, 40]. While decompressing the portal venous system does not reverse cytopenias, reducing stem cell demand by replacing an injured organ can do so, as in the case of liver transplantation [10, 22]. Similarly, reducing auto injury in SLE with bone marrow transplantation can reverse cytopenias [58].

If stem cell resources are finite, then non-repair uses for stem cells might be downregulated in the event of injury, an active process such as Dameshek envisioned. G-CSF does inhibit bone production while mobilizing stem cells from bone marrow [31]. This suggests that repair efforts take priority, and thus it would be reasonable to expect downregulation of blood production, as Dameshek suggested. Studies of sera from patients with liver disease and those with SLE indicate that downregulation of hematopoiesis may occur [32, 33].

The above is neither intended to provide a comprehensive explanation of the cytopenias of liver disease and SLE, nor to try to simplify the interactions among bone marrow, spleen, and blood components. On the contrary, an active process would add complexity by suggesting that there is at least some contribution by a process not currently recognized. How much of a contribution to cytopenias an active suppression of hematopoiesis might make is unclear. Cytopenias are most likely multifactorial, and downregulation of hematopoiesis would more likely have identifiable effects in chronic rather than acute injury. Acute malaria causes liver injury with increased serum G-CSF, yet hematologic abnormalities can be present within days of onset of symptoms, sooner than would be expected due to an altered rate of production [36, 60].

Cytopenias in SLE are likely to be particularly complex in nature. There are more than 100 autoantibodies reported in SLE, including autoantibodies to platelet glycoproteins thought to result in platelet destruction [61, 62]. In addition, Su et al. have reported that, “leucocyte apoptosis is significantly higher in SLE patients and correlates well with the levels of several autoantibodies” [63].

Although Dameshek may be correct in that there is an active process suppressing hematopoiesis, this does not mean that Doan’s theory of sequestration is entirely incorrect. While evidence that portal pressure affects spleen size is lacking, blood components can be trapped in the spleen. Note that in primary immune thrombocytopenia, tagging platelets with Indium 111 often demonstrates platelet sequestration in the spleen, and that in this disease splenectomy can be effective in relieving thrombocytopenia [64].

On the whole, the current evidence does support Dameshek’s theory that there is suppression of hematopoiesis, at least in some instances.

### Clinical implications

The existence of an active signaling mechanism directing allocation of stem cell resources would raise the possibility for errors of that system. For example, over-suppression of hematologic production might account for some cases of cytopenias, notably those that occur following viral hepatitis [65].

Knowledge that a signaling mechanism may be in action directing the flow of stem cell resources could aid in the work-up of anemias. Evaluation of anemia in patients with SLE, liver disease or other chronic illness could include evaluation of stem cell demand, such as obtaining serum levels of IL-23 and G-CSF.

A signaling mechanism could also provide an opportunity for therapy. Rather than treating cytopenias in patients with liver disease by embolizing portions of the spleen, clinicians could seek agents that block the routing of stem cells to repair. This would have the disadvantage of reducing repair efforts, but would also offer the potential to lessen cytopenias without permanent damage to the spleen.

## Abbreviations

ALT: Alanine transaminase
G-CSF: Granulocyte-colony stimulating factor
HCC: Hepatocellular carcinoma
IL-17: Interleukin 17
IL-23: Interleukin 23
INR: international normalized ratio
MELD: Mayo end-stage liver disease score
SLE: systemic lupus erythematosus
TIPS: transjugular intrahepatic portosystemic shunt
TPO: thrombopoietin
UNOS: United Network for Organ Sharing
